# How Pharmaceutical Residues Occur, Behave, and Affect the Soil Environment

**DOI:** 10.3390/jox14040076

**Published:** 2024-10-01

**Authors:** Gabriel Pérez-Lucas, Simón Navarro

**Affiliations:** Department of Agricultural Chemistry, Geology and Pedology, School of Chemistry, University of Murcia, Campus Universitario de Espinardo, E-30100 Murcia, Spain; gpl2@um.es

**Keywords:** emerging pollutants, pharmaceuticals, plant uptake, soil behavior, soil remediation

## Abstract

Many pharmaceuticals (PhMs), compounds for the treatment or prevention of diseases in humans and animals, have been identified as pollutants of emerging concern (PECs) due to their wide environmental distribution and potential adverse impact on nontarget organisms and populations. They are often found at significant levels in soils due to the continuous release of effluent and sludge from wastewater treatment plants (WWTPs), the release of which occurs much faster than the removal of PhMs. Although they are generally present at low environmental concentrations, conventional wastewater treatment cannot successfully remove PhMs from influent streams or biosolids. In addition, the soil application of animal manure can result in the pollution of soil, surface water, and groundwater with PhMs through surface runoff and leaching. In arid and semiarid regions, irrigation with reclaimed wastewater and the soil application of biosolids are usual agricultural practices, resulting in the distribution of a wide number of PhMs in agricultural soils. The ability to accurately study the fate of PhMs in soils is critical for careful risk evaluation associated with wastewater reuse or biosolid return to the environment. The behavior and fate of PhMs in soils are determined by a number of processes, including adsorption/desorption (accumulation) to soil colloids, biotic (biodegradation) and abiotic (chemical and photochemical degradation) degradation, and transfer (movement) through the soil profile. The sorption/desorption of PhMs in soils is the main determinant of the amount of organic chemicals taken up by plant roots. The magnitude of this process depends on several factors, such as crop type, the physicochemical properties of the compound, environmental properties, and soil–plant characteristics. PhMs are assumed to be readily bioavailable in soil solutions for uptake by plants, and such solutions act as carriers to transport PhMs into plants. Determining microbial responses under exposure conditions can assist in elucidating the impact of PhMs on soil microbial activity and community size. For all of the above reasons, soil remediation is critical when soil pollutants threaten the environment.

## 1. Introduction

Since the beginning of this century, a great body of qualitative and quantitative research has been conducted worldwide to investigate the incidence and fate of emerging pollutants (EPs), also known as pollutants of emerging concern (PECs), in the environment as a result of point and diffuse pollution [1]. Recent studies have focused on the presence of a variety of identified anthropogenic compounds in addition to microorganisms in the environment from urban wastewater (WW), industrial effluents, hospitals, livestock, and agriculture [2]. Their occurrence in and adverse effects on terrestrial and aquatic ecosystems and human health are of current concern to scientists because of uncertainties surrounding their ecotoxicity [3,4]. A review of the literature obtained from the Web of Science™ (WoS), managed by Clarivate Analytics (Philadelphia, PA, USA), using the following search terms, “emerging pollutants” (title) OR “emerging contaminants” (title) AND “environment” (topic) AND “2010–2024” (year published), revealed more than 2600 publications, with a clear trend of exponential growth in recent decades.

With the development of analytical techniques, more and more environmental EPs are being identified in addition to the well-known ones; these techniques, mainly gas (GC) and liquid (LC) chromatography coupled to mass spectrometry (MS), allow for the detection of very low levels (ng L^−1^/ng kg^−1^) in environmental matrices, although their toxicity, environmental occurrence, and characteristics are still less known than those of conventional pollutants [5,6,7,8,9,10,11].

EPs are certainly not new substances. They are chemicals and/or microorganisms, often long present in the environment. However, their presence and importance are only now being understood. EPs can be defined as new chemicals, involving their transforming intermediates, that have not undergone a regulatory evaluation and whose environmental and human health effects are not well understood. More than 30,000 chemicals found in everyday products, many of which are recognized by the European Chemicals Agency (ECHA) based on the substances listed under the European Regulation on the Registration, Evaluation, Authorization and Restriction of Chemicals (REACH) [12], are catalogued as potential EPs due to their environmental release. These include pesticides, pharmaceuticals, personal care products, illicit drugs, lifestyle compounds, industrial compounds and byproducts, flame retardants, microplastics, and disinfection byproducts, and, more recently, microorganisms like SARS-CoV-2 have been found in WW worldwide [13,14,15,16].

**Figure 1:**
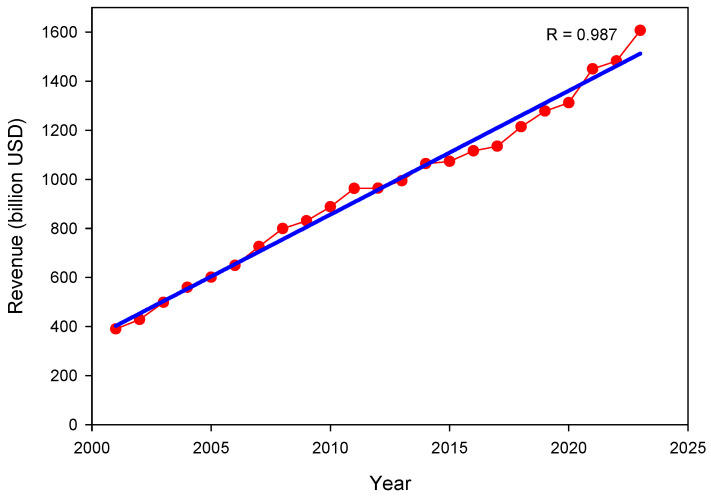
Worldwide pharmaceutical market sales (2001–2023). Red points are values (billion USD per year) and blue line is regression line.

Pharmaceuticals (PhMs) are products containing active ingredients at specific dosages that can be used for health care purposes. The population profile of most countries is aging, as the proportion of the world’s population aged 65 or over is steadily increasing, which is a major driver of the PhM market. The research, development, production, and distribution of medicines are the responsibilities of the pharmaceutical industry. The market has experienced significant growth since the beginning of the century, with a linear increase (R = 0.99) and revenues of USD 1.61 trillion worldwide in 2023, and it is expected to grow at a compound annual growth rate (CAGR) of 7.6% from 2023 to 2030 [17] (Figure 1).

Through the improvement of science and the development of research technologies, the pharmaceutical industry has achieved many benefits in protecting human and animal health and life. However, the many unresolved problems of drug residues in the environment should also be considered when discussing achievements in pharmacotherapy [18,19,20,21,22]. In particular, PhMs are often found at significant concentrations in soils due to the continuous release of effluent and sludge from WWTPs, which is known to occur faster than their degradation [23,24,25,26,27,28,29]. Since the 1990s, a number of PhMs have been reported to have adverse effects on organisms such as fish and algae in the environment [30]. After ingestion by humans and animals, some of them are excreted unaltered and can therefore reach WWTPs via the sewerage system [31,32]. In addition, most PhMs are metabolized in humans and animals. The metabolites are mostly excreted and subsequently enter the environment directly or via WWTPs [33]. The levels of PhMs in the environment have only recently begun to be monitored and recognized as potentially dangerous to ecosystems, and, in recent years, PhMs as environmental contaminants have become a major public health concern. In this context, Directive 2013/39/EU [34] has identified so-called priority substances, the emissions of which have to be reduced or phased out, and it has established the need for the creation of a list of EPs to be monitored. As a result, compounds such as some PhMs (macrolide antibiotics, diclofenac, sulfamethoxazole, and venlafaxine) and other EPs have been included in various implementation decisions (2015/495/EU, 2018/840, and 2020/1161) due to their prevalence, persistence, and lack of regulation. All of this aims to meet goals such as the zero emissions target announced in the European Green Deal [35] and is linked to the Sustainable Chemicals Strategy [36]. In the context of this initiative, PhMs are highlighted in the Strategic Approach on Pharmaceuticals in the Environment [37] and the Pharmaceuticals Strategy for Europe [38], both of which highlight the environmental and potential health impacts of pollution from PhM residues and list a number of actions to address these challenges, including the following six: (i) raise awareness and promote prudent use, (ii) improve training and environmental risk assessment, (iii) collect monitoring information, (iv) promote greener pharmaceutical development, (v) reduce manufacturing emissions, and (vi) reduce waste and improve WW management. More recently, a number of PhMs used as painkillers, anticonvulsants, and antibiotics were included in the European Commission’s October 2022 proposal to revise the list of priority pollutants in surface water [39]. These proposals aim to counteract the negative impacts of PhMs on the environment and cover all phases of the life cycle of PhMs, from design and production to use and disposal.

Therefore, further action is needed to prevent pollution. It is also necessary to take measures to clean up and remove pollution to improve its subsequent reuse while protecting the health of citizens. The treatment of contaminated WW (from agricultural, industrial, and urban areas) by conventional WWTPs may be inadequate to reach the quality levels required by law for some of the most persistent pollutants [40]. This fact is evidenced by numerous monitoring reports of WW effluent conducted worldwide, where a wide variety of PhMs have been detected [10,31,41,42,43,44]. To supplement water resources, reclaimed water is increasingly being used. However, this water has a complex matrix that contains EPs that are released into the soil when it is used for irrigation.

Water scarcity and the unequal geographical distribution of rainfall are issues of concern in arid and semiarid areas such as the Mediterranean basin, where water management strategies advocate reusing treated WW effluent in agriculture due to climate change. Many compounds enter the receiving environment with or without modification due to the wide variety of EPs entering WWTPs. The agro-environment (soil and crops) is exposed to WW and, through its discharge into water bodies, to PhMs. Many of these are still unknown and have not been evaluated [22,24]. In order to address this concern, the EU has focused on this issue by revising the minimum requirements for water reuse in the context of integrated management under Regulation 2020/741/EU on the minimum requirements for water reuse [45]. The goals are to ensure that recycled water is safe for crop irrigation, promote a circular economy, and support resilience to climate change by addressing water scarcity and related pressures on water resources. Thus, where essential and applicable, water quality requirements and monitoring will include, in addition to BOD_5_, TSS, turbidity, *E. coli*, *Legionella* spp., intestinal nematodes, and other factors, the monitoring of PhMs and other EPs, such as heavy metals, disinfection byproducts, pesticides, and microplastics, to ensure the security of the environment and animal/human health. Although conventional WWTPs act as a primary barrier against environmental pollution by EPs, they are not designed to eliminate them at low levels or are unsuccessful in removing them [46]. Therefore, many countries around the world are investigating the upgrading of WWTPs with new (advanced) technologies to achieve the removal of EPs and protect the environment [42,44,47,48,49,50,51,52,53,54,55]. As a result, growing government concern about the presence of PhMs and other EPs in WW is leading to the development of new policies that will have a significant impact on the design and operation of wastewater treatment plants in the years to come.

Although PhMs are generally present at low environmental concentrations, it remains uncertain whether their presence in terrestrial and aquatic environments may undesirably affect wildlife and humans. Therefore, to obtain better knowledge of their impact on the environment and human health, information on their occurrence, behavior, and fate in the environment must be studied in detail. Therefore, the occurrence, environmental fate, and impact of PhMs on the soil are the focus of this review.

## 2. Methodology

The Science Citation Index Expanded (SCIE) of the Web of Science Core Collection (WoSCC) database was used for this review, being the world’s oldest bibliographic source and the most comprehensive and widely used source for evaluating and analyzing research [56]. The scientific literature published in the last 15 years was searched and analyzed during May 2024. Before the process began, and as the extracted information was reviewed, the following questions were asked and considered: (i) What are the sources of PhMs in the environment? (ii) How do PhMs occur in the soil environment, and how do they behave? (iii) How do PhMs affect soil health? (iv) How can PhM-polluted soils be remediated? The analysis included records resulting from a systematic search for documents containing a combination of the following terms: “pharmaceuticals” (title) AND “soil” (title) AND “2010–2024” (year of publication) due to the exponential growth of publications during this period. Our search identified 219 publications containing 5304 cited articles that were considered eligible for inclusion in this review. This analysis was extensively standardized to minimize the risk of bias. In addition, there was a search for other relevant studies published by international organizations and expert working groups.

## 3. Sources of PhMs in the Environment

PhMs released into the environment can be separated into two broad groups: veterinary (VPhMs) and human (HPhMs) PhMs. VPhMs are used worldwide to treat diseases and safeguard animal health, and the type of compound used depends on the animal sector and specific region [57]. Common classes of veterinary medicines found in animal products in Europe include antimicrobials, anti-inflammatories, growth promoters, antiparasitics and insecticides, and tranquilizers [58,59]. The land application of animal manure can lead to the VPhM pollution of soil, surface water, and groundwater via surface runoff and leaching [60,61,62,63,64,65]. However, HPhMs are in widespread use and prescription in human medicine worldwide. They represent a wide range of therapeutic classes. Data extracted from different European countries indicate that analgesics and anti-inflammatory drugs (acetaminophen, acetylsalicylic acid, ibuprofen, dicoflenac, etc.), antibiotics (macrolides, penicillins, quinolones, tetracyclines, and others), antiepileptics (carbamazepine), β-blockers (atenolol, propanolol, etc.), hormones (progesterone, testosterone, and 17α-ethinylestradiol), lipid regulators (lovastatin, clofibrate, etc.), selective serotonin reuptake inhibitors (SSRIs) (fluoxetine and paroxetine), illicit drugs (cocaine, cannabinoids, amphetamines, opioids, etc.), and others are the compounds sold in the greatest quantities [18,59,66]. Table 1 shows the chemical structures and physicochemical properties of the major HPhMs used [67].

**Figure 2:**
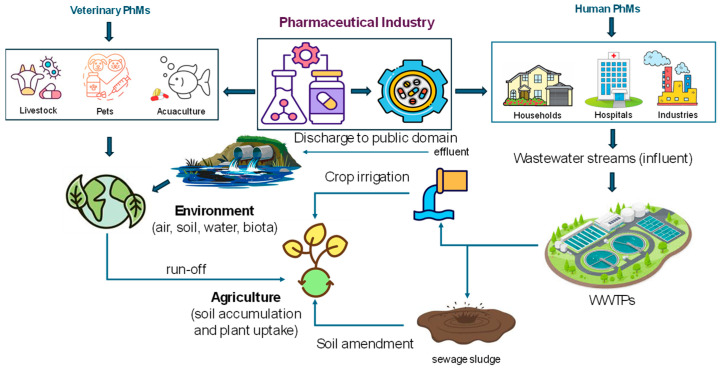
Main sources of VPhMs and HPhMs in the environment.

The main sources of both groups of PhMs in the environment are shown in Figure 2. The excretion of urine and feces from medicated animals and the application of contaminated manure to agricultural soils are important pathways for VPhMs to enter the environment. The residues of VPhMs in soils, plants, and soil organisms can subsequently enter the food chain [68]. A number of comprehensive studies have examined the fate and effects of VPhMs in the environment, either in a regional or national context or from a European or global point of view [69]. The results show that the release of VPhMs into the environment varies between livestock sectors due to variations in the use and excretion of VPhMs and manure production. The main conclusion of these studies is that information on veterinary medicines is available for the identification of environmental risks. However, quantitative information about administered veterinary drugs is very limited and needs to be urgently addressed. The main sources of HPhMs in the environment are the discharge of WW from industrial processes, the improper disposal of unused or expired compounds, accidental spills during production or distribution, the discharge of treated WW into the aquatic environment, the use of treated WW for the irrigation of crops, and the use of SS as fertilizer (organic amendment) in agricultural fields [66]. The disposal of incinerated pharmaceutical waste in landfills can also cause environmental pollution. However, this is changing because of stricter regulations such as the EU Landfill Directive [70] and subsequent regulations [71]. Reducing the amount of waste going to landfills is a key objective of EU waste policy. The landfill rate is decreasing (from 23% to 16% overall between 2010 and 2020 in the EU-27). This is despite the fact that the total amount of waste generated continues to increase. In addition, the amount of waste that went to landfills in 2020 is 27% lower than that in the same period in previous years. This is equivalent to 106 kg of waste per EU citizen per year. Relatively good progress has been made in diverting waste from landfills for some waste streams, such as (mixed) household and similar waste. However, the amount of sorted residual waste going to landfills has doubled since 2010.

Abdallat et al. [72] showed that, on all farms using a drip irrigation system with different WWTP effluents, carbamazepine concentrations were greater in the topsoil layers (0 to 20 cm) than in the root zone (20 to 40 cm). In plants, especially during the olive growing season, carbamazepine was detected only in olives and not in twigs or leaves, highlighting a high rate of plant uptake. Furthermore, the fruits, leaves, and stems of plants left on the farm after harvest are usually eaten by cattle. This means that they enter the human food chain. Other results showed the ubiquitous presence of several PhMs in the effluents of WWTPs used for irrigation, although the pattern of concentrations in water did not resemble the pattern of levels in soil and plants, as only paracetamol, nicotine, ibuprofen, and carbamazepine were detected in maize grains at rather low levels, confirming the restricted potential for the uptake of PhMs by maize [73]. Many studies have been conducted, especially at lab scale, showing that the transfer of a pollutant from water and/or soil to plants depends on various factors, such as the physicochemical properties of the soil (pH, clay, and organic matter content), the plant species, and the properties of the pollutant (ionization, water solubility, etc.) itself [74,75]. Despite the importance of this, there is a need for studies of the plant uptake of micropollutants under real agricultural practices, where pollutants are present in the soil environment as multicomponent combinations. Christiou et al. [24] indicated that the concentrations of diclofenac, sulfamethoxazole, and trimethoprim in soil and tomato fruits varied depending on the qualitative characteristics of the applied treated WW and the irrigation time. The total concentrations of PhMs detected by Biel-Maeso et al. [25] in surface soil samples after irrigation with WW effluent ranged from 2 to 15 ng g^−1^, with anti-inflammatories and analgesics being predominant (maximum = 10 ng g^−1^), followed by psychiatric drugs and antibiotics (maximum = 3.8 ng g^−1^ and 5.4 ng g^−1^, respectively). WW effluents and irrigated soils showed similar composition patterns, with active ingredients such as diclofenac and hydrochlorothiazide being predominant. In addition, they were also detected in soil samples (150 cm depth), suggesting that these compounds are leached in association with heavy rainfall episodes. In addition, maximum levels (up to 14 ng g^−1^) were measured in colder months, indicating that their occurrence in soils is also influenced by temperature, with greater persistence at lower temperatures. Another study showed that the highest amounts of PhMs discharged via secondary effluent were related to an antihypertensive drug and several beta-blockers and analgesics/anti-inflammatories, while antibiotics and some psychiatric and analgesic/anti-inflammatory drugs posed the highest risk [41]. These results can help scientists and managers plan measures to reduce the impact of the discharge of treated municipal WW on surface waters and/or soils.

The European policy directly aims to increase the agricultural reuse of sewage sludge (SS) in soil to improve its fertility. However, the long-term effects on soil properties are still unknown. The agronomic and environmental soil effects after 17 years of organic amendment with SS used in daptomycin production were evaluated by Cucina et al. [76]. They showed a positive agronomic potential, improved soil organic matter quality, increased soil humified organic matter, and increased plant nutrients. However, with long-term agricultural reuse, environmental risks have been associated with increases in some heavy metals (Zn, Hg, and Cu) and exchangeable Na. However, the SS generated in WWTPs and managed for agriculture poses the risk of spreading all the micropollutants that it contains [77]. The factor limiting the agricultural application of SS is sanitary pollution, where PhMs are a significant concern, and this may affect the restriction of their use as fertilizers. Mejías et al. [28] recently reported that antibiotics, antimicrobials, antidepressants, antidiabetics, and nonsteroidal anti-inflammatory drugs were the most abundant PhMs found in SS. Overall, their levels decreased during sludge stabilization, especially during anaerobic digestion and composting. The sorption of PPCPs to SS is strongly correlated with the physicochemical properties, the composition of the sludge matrix, and the operational and environmental conditions. The total concentrations of 38 selected pharmaceuticals from seven different therapeutic classes (antibiotics, anticancer agents, analgesics, anti-inflammatories, beta-blockers, lipid regulators, and psychotropics) monitored in the anaerobically treated sludge of urban WWTPs in Turkey ranged from 280 to 4898 µg kg^−1^ dm, with azithromycin and clarithromycin being the most abundant compounds [78]. A study by Verlicchi and Zambello [79] provided a snapshot of the incidence of selected compounds in primary, secondary, mixed, digested, conditioned, composted, and dry sludge from municipal WWTPs fed primarily with municipal wastewater and in sludge-amended soil. The study concluded that the most critical compounds found in sludge-amended soil were caffeine, ciprofloxacin, 17β-estradiol, ofloxacin, tetracycline, triclosan, and triclocarban. Some studies have shown that the levels of PhMs in soil are clearly correlated with the maturity of the sludge used [26]. Martín et al. [80] evaluated the distribution and ecotoxicological risk of 16 PhMs belonging to seven therapeutic groups (two antibiotics, five anti-inflammatory drugs, one b-blocker, one antiepileptic drug, one nerve stimulant, two lipid regulators, and four estrogens) in SS from WWTPs. Only 3 of the 16 pharmaceuticals were never detected in the SS, while 11 of the investigated PhMs were still found in the compost. 17β-Estradiol posed the highest ecotoxicological risk in the digested sludge and compost. High levels of ciprofloxacin, norfloxacin, and ofloxacin were found in SS [81]. In addition, these antibiotics were detected long after the application of the SS, which suggests the persistence of these compounds in soil. Moreover, in soils treated with composted sludge, the concentrations of this group of antibiotics were clearly lower [82]. Martín et al. [83] evaluated the contamination of PhMs in digested sludge and compost, with the maximum levels found in digested sludge corresponding to caffeine (up to 115 ng g^−1^), ibuprofen (45 ng g^−1^), and carbamazepine (9.3 ng g^−1^). The concentrations determined in the compost were even lower than those in the digested sludge, and no compounds were detected in the sludge-amended soils, possibly because of the dilution effect after sludge application to the soil. The presence of different antibiotics (amoxicillin, cefuroxime, ciprofloxacin, clarithromycin, levofloxacin, lincomycin, norfloxacin, sulfadiazine, and trimethoprim) in the SS of different WWTPs in Galicia (NW Spain) was studied by Barreiro et al. [84]. The results showed that almost all SS samples contained antibiotics, with ciprofloxacin and levofloxacin being the most abundant (maximum values of 623 and 893 ng g^−1^, respectively). Treatment with SS resulted in a significant reduction in the number and concentration of antibiotics. In 12% of the soil samples in which the sludge was applied, some antibiotics were detected, but they were always present at low levels. With regard to crops, no antibiotics were found in the roots, stalks, leaves, or grains of maize or in grapes sampled in vineyards. Overall, sludge treatments could have a large impact on soil pollution. Once applied to soil, the agronomic benefits will depend on the soil and sludge quality. The risks associated with the ecotoxicity of PhMs found in sewage sludge are low. However, they are not insignificant [26].

## 4. Behavior and Fate of PhMs in the Soil Environment

**Figure 3:**
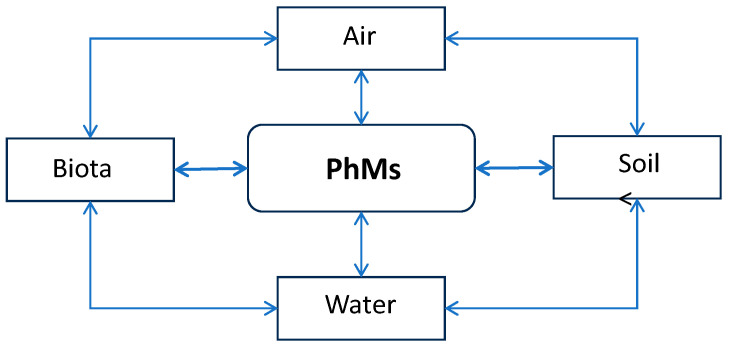
Environmental compartmentalization of PhMs.

PhMs can enter the soil environment from the pharmaceutical industry (point sources), from the application of animal manure and SS as fertilizer, or from irrigation with contaminated water (nonpoint sources) [85]. The occurrence of PhMs in the environment from veterinary and human consumption has been the subject of increased scientific attention in recent decades due to concerns about their combined environmental effects on aquatic and terrestrial environments and, to some extent, human health [86,87]. For an accurate risk assessment associated with WW reuse or biosolids recycling to the environment, the ability to accurately determine the fate of PhMs in soil is needed. The natural affinity of one of the four environmental compartments (soil, water, air, or biota) determines the environmental behavior and fate of PhMs (Figure 3).

The tendency of a compound to move from one compartment to another is often referred to as compartmentalization. To understand the behavior of PhMs in the environment and their ultimate fate, it is necessary to know certain information about their identity and chemical composition, their physicochemical properties, and the environmental characteristics of the site where they have been released. Some of these physicochemical properties are summarized in Table 2.

**Figure 4:**
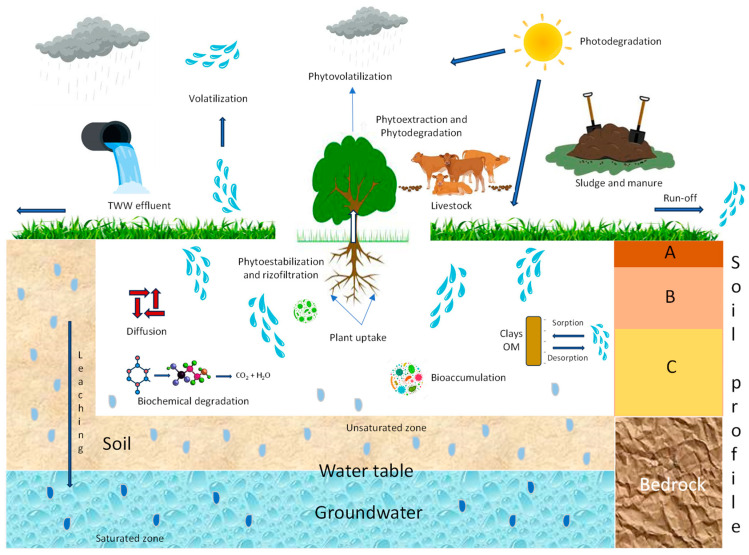
Schematic drawing of the behavior and fate of PhMs in soil.

These parameters are well known and are used to predict the environmental fate of a given compound and to estimate its persistence, understood as “the tendency of a given PhM to retain its structural properties unchanged for a given period of time in the environment in which it is distributed and/or transported”. Once incorporated into the soil, PhMs enter a dynamic ecosystem and begin to move within it, degrading “in situ”, remaining in it with their original structure or being degraded over a variable period of time (Figure 4).

PhM concentrations may be virtually constant for a period of time, followed by a monophasic or biphasic decrease. There are three phases involved in the disappearance of a PhM in soil: (i) the lag phase, which is a short period of time during which the ingredient is maintained at a certain concentration; (ii) the dissipation phase, which is relatively rapid in terms of disappearing from the soil; and (iii) the persistence phase, which is responsible for the persistence of the PhM and is expressed in hours, days, weeks, months, and even years. The half-life (*t*_1/2_), defined as “the time required for half the amount of PhM initially present or deposited in the soil to disappear”, is the most commonly used term to express persistence. In some cases, it is more appropriate to use the disappearance time expressed as DT_50_, DT_75_, and/or DT_90_, which specify “the time required for 50, 75 and/or 90% of the initial concentration of the PhM present or introduced in soil to disappear”. According to Gavrilescu [89], a compound with a half-life (*t*_1/2_) greater than 100 days is considered persistent, while nonpersistent compounds have a *t*_1/2_ < 30 days. Therefore, those with 30 > *t*_1/2_ < 100 days are classified as moderately persistent. Kodešová et al. [90] studied the persistence of different groups of PhMs, such as antibiotics (clindamycin, clarithromycin, sulfamethoxazole, and trimethoprim), beta-blockers (atenolol and metroprolol), and psychotropics (carbamazepine), in 13 different soils. Carbamazepine had the greatest persistence in soil, followed by clarithromycin, trimethoprim, metroprolol, clindamycin, sulfamethoxazole, and atenolol. The half-life, which is related to soil properties, reflects the sorption process of PhMs on soil particles and increases with an increasing soil sorption capacity.

**Figure 5:**
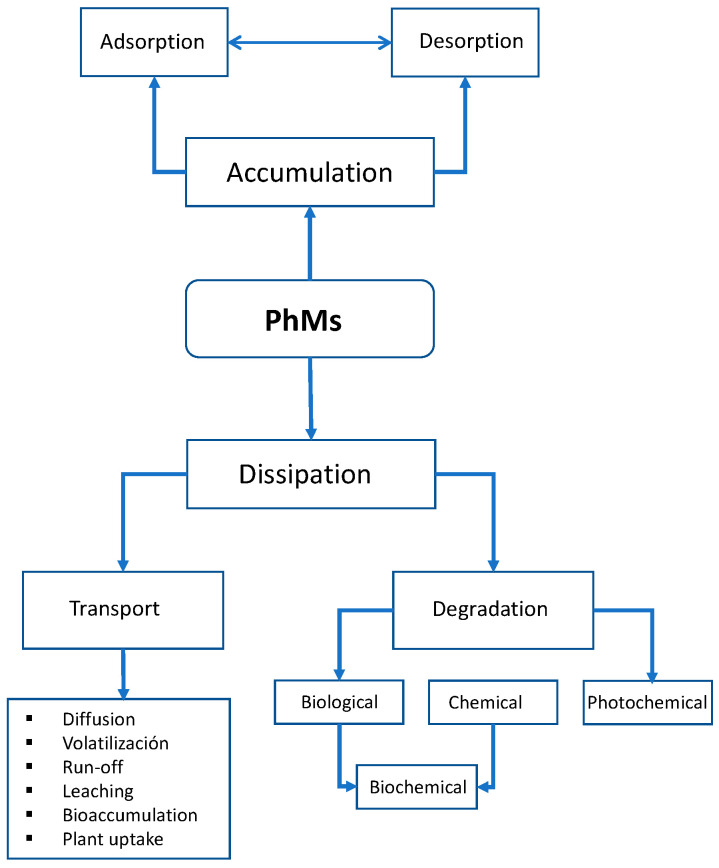
Processes involved in the fate of PhMs in soil.

The behavior and fate of PhMs in soils are governed by a number of processes [91]. These include sorption (adsorption/desorption) to organic and inorganic colloids, biotic (biodegradation) and abiotic (chemical and photochemical degradation) degradation, and transfer (movement) through the soil profile (a vertical section showing its horizons and the primary material and hence availability for plant uptake) [92,93]. Soil–pharmaceutical–plant interrelationships are quite complex (Figure 5). Colloidal adsorption/desorption and degradation dominate this dynamic process (inactivation, loss, and transformation), which involves various physical, chemical, and microbiological processes, all of which are interrelated and responsible for their behavior and ultimate fate [94]. It is possible to predict the behavior of PhMs in soil and their possible toxicological effects with remarkable reliability based on physicochemical properties and adsorption, degradation, and mobility data obtained in the laboratory.

### 4.1. Adsorption/Desorption

Sorption is one of the key processes affecting the fate, mobility, migration, and bioavailability of PhMs in soil. It depends on several factors such as the physicochemical properties of the soil and PhMs, soil reaction, surface activity, temperature, and moisture [94]. In general, adsorption can be defined as the enrichment of molecules, atoms, or ions near an interface. PhMs (adsorbates) that can be retained in soil colloids (adsorbents) are subject to this concept. Due to the innumerable negative charges of both colloids on the natural pH of soil, the colloidal fractions of soil, both inorganic (clays) and organic (humus), play a fundamental role in this process [95].

Two types of forces are involved in this process: physical adsorption (physisorption), involving van der Waals interactions, and chemical adsorption (chemisorption), involving chemical bonds between adsorbed molecules. The distribution of a PhM between the water and soil compartments depends on the properties of the compound and the matrix and may also be influenced by external factors such as temperature and soil moisture. The relationship between the concentrations of a substance in the solid and liquid phases is called the coefficient of distribution (*K_d_*) and is directly proportional to the solubility of the PhM in water and inversely proportional to the clay and OM content in the soil (Equation (1)):

(1) $$k_{d}=\frac{C_{a}}{C_{e}}$$

where *C_a_* is the amount of PhM adsorbed per unit mass of adsorbent (M/M), and *C_e_* is its concentration in solution (M/V).

Batch experiments are commonly used for the direct measurement of *K_d_* (mL g^−1^) [96,97]. A mass (g) of soil is mixed with a volume (mL) of water or another medium, such as aqueous 0.1 M CaCl_2_ (to minimize the disturbance of the soil mineral balance). To give an initial concentration of the chemical in the liquid phase, a mass (g) of a PhM is added to the slurry (or added to a phase before mixing). The slurry is then gently mixed to minimize disturbance to the soil structure. This is typically performed for a period of between 2 and 48 h (usually 24 h). This is followed by an analysis of the equilibrium concentration *C_e_* of the PhM in the aqueous solution. High *K_d_* values (>100) indicate that the majority of PhMs are adsorbed on the soil surface at any given time and are therefore less likely to be mobile in soil, but this is not an indication of the strength (reversibility) of this sorption. *K_d_* is often standardized to the organic content (*OC*) of the soil to determine the organic carbon–water partitioning factor, *K*_OC_ (mL g^−1^), which is typical of many current protocols (Equation (2)):

(2) kOC=kdOC×100

where *K_d_* is the coefficient of distribution, and *OC* is the organic carbon content (%).

This approach was originally developed for hydrophobic compounds. However, it is not clear whether such normalization is appropriate for ionizable PhMs. The consideration of soil pH may be more relevant for such normalization, especially considering the potential effects of the *pK_a_* of a PhM on its potential ionization and subsequent sorption. Karickhoff et al. [98] showed that there is a linear correlation between the partition coefficient and soil organic carbon content. *K*_OC_ is linearly correlated with the octanol–water partition coefficient (*K*_OW_), which is an indicator of the degree of affinity of PhMs for water (low value) or soil (high value). The molecule will not be bioavailable if high adsorption exists (chemisorption). Thus, its biological activity will be reduced. In addition, it is not biodegradable, which increases its persistence in soil, and its mobility will be greatly reduced, so the possibility of groundwater contamination will be minimal. However, the compound may desorb and return to the soil with the associated biocidal risk when the soil conditions change (moisture, temperature, etc.). *K_d_* values are often determined over a range of concentrations at a constant temperature. The resulting plot, the relationship between the adsorbed concentration (*C_a_*) and the equilibrium concentration of the compound (*C_e_*) at a constant temperature, is called the adsorption isotherm.

The plant uptake of PhMs is generally reduced by soil sorption. This is especially true for strong hydrophobic or positively charged chemicals [75,99,100]. Lin and Gan [93] showed that the degradation rate of PhMs is influenced by the presence of microorganisms and the aerobic conditions in the soil, by the soil type, and by the properties of the PhM itself, indicating that naproxen and trimethoprim showed moderate/strong sorption, while the sorption of ibuprofen, diclofenac, and sulfamethoxazole was negligible in the soils studied, which could increase their mobility in soils and cause groundwater pollution. The greater persistence of ketoprofen in OM-rich soils may be explained by the reduced availability of this compound due to increased adsorption in this soil type. The relationship between the sorption capacity of the soil and the half-life of the compound was clearly demonstrated by the fact that the half-life of ketoprofen was the longest in silt loam and the shortest in loamy sand [101]. The results of another study showed that levonorgestrel (a synthetic progesterone used as an active ingredient in hormonal contraception) can be highly absorbed in soil, mainly by binding to *OC* [102]. The adsorption of PhMs depends on the activity of microorganisms, in addition to their hydrophobic properties and electrostatic interactions with soil particles. The acidic compounds, including diclofenac, acetylsalicylic acid, ketoprofen, ibuprofen, naproxen, and indomethacin, with *pK_a_* values ranging from 4 to 5, similar to those of clofibric acid and bezafibrate (*pK_a_* 3.6), exist in ionic forms at neutral pH and have a low tendency to adsorb to soils, although the sorption of these compounds in soil increases at lower pH values. At neutral pH, negatively charged PhMs are mainly in the liquid phase [103]. Paz et al. [104] studied the sorption of two highly persistent anticonvulsants, carbamazepine and lamotrigine, and two of their metabolites (EP-CBZ and DiOH-CBZ) by soils and found that it was mainly determined by the soil OM content. The compounds’ sorption affinities to soils followed the order of lamotrigine > carbamazepine > EPCBZ > DiOH-CBZ. The results of lysimeter assays were consistent with the results of the batch experiments and showed the accumulation of lamotrigine and carbamazepine in the OM-enriched topsoil layers. Similar results were found by Thiele-Bruhn and Zhang [105], who worked with sulfadiazine, caffeine, and atenolol in arable Cambisol topsoil after the addition of manure. According to Kodešová et al. [106], pedotransfer rules for predicting the sorption coefficients of various PhMs include hydrolytic acidity (sulfamethoxazole), *OC* content (trimethoprim and carbamazepine), base cation saturation (metoprolol and atenolol), exchangeable acidity and clay content (clindamycin), and soil active pH and clay content (clarithromycin). Pedotransfer rules, which predict Freundlich sorption coefficients, can be used to predict PhM mobility in soils with similar properties. The calculated sorption coefficients can be used to assess potential groundwater contamination in conjunction with PhM half-lives and other inputs (e.g., soil hydraulic, geological, hydrogeological, and climatic inputs).

Kočárek et al. [107] evaluated the sorption capacities of four PhMs (atenolol, carbamazepine, sulfamethoxazole, and trimethoprim) added in solute mixtures to soils taken from different horizons of three soil types. The sorption affinity of these compounds for carbamazepine (neutral form) and sulfamethoxazole (partially negatively charged and neutral) decreased with soil depth (i.e., with the soil OM content). The sorption affinity of the compounds was not depth-dependent for atenolol (positively charged) or trimethoprim (partially positively charged and neutral). Batch sorption experiments of ketoprofen, atenolol, and carbamazepine on biochar-amended soils conducted by Wu and Bi [108] showed that the sorption affinity decreased in the order of cationic atenolol > neutral carbamazepine > anionic ketoprofen due to the electrostatic attraction of atenolol to the amended soils. A thermodynamic study carried out to investigate the adsorption behavior of three ionizable compounds present in different forms, propranolol (cationic), sulfisoxazole (anionic), and sulfaguanidine (neutral), on soil under different temperature conditions revealed that the sorption of propranolol is exothermic, spontaneous, and enthalpy-driven, while the sorption of sulfaguanidine is endothermic, spontaneous, and entropy-driven, and the sorption of sulfisoxazole is endothermic, spontaneous only above 303.15 K, and entropy-driven [109]. The sorption/desorption behavior of four commonly consumed anti-inflammatory drugs (naproxen, diclofenac, ibuprofen, and ketoprofen) was investigated by Zhang et al. [110] in a loam-textured soil exposed to either a single compound or a mixture of the four compounds. The results showed that the proportion adsorbed on the soil in the mixture–compound system was 72% and 45% for diclofenac and ibuprofen, respectively, and it was slightly different from the adsorption of the individual compounds.

Several models have been developed to estimate the soil sorption of organic chemicals, including ionizable compounds [111,112], although the pertinency of these models to PhMs has not been significantly investigated. The results show that sorption coefficients for PhMs in soil can vary over a range of many orders of magnitude (e.g., 0.09 for sulfameter < *K_d_* < 1,277,873 for ciprofloxacin mL g^−1^), and sorption coefficients for a single PhM can vary by up to three orders of magnitude between different soil types (e.g., *K_d_* values for ciprofloxacin range from 726.8 to 1,277,873 mL g^−1^). Li et al. [113] generated a high-quality data set on the sorption of 21 PhMs in different soil types and used these data to evaluate existing models and to develop new improved models. They found *K_d_* values ranging from 0.2 (antipyrine) to 1249 mL g^−1^ (perphenazine). A principal component analysis (PCA) revealed that the sorption of the PhMs under study was controlled by hydrophobic forces, as well as electrostatic interactions and a number of soil parameters.

### 4.2. Degradation

The residues of PhMs found in soils can lead to significant bioaccumulation with adverse consequences on soil organisms, crops, and even humans through dietary consumption [114]. Laboratory studies have shown that the degradation rates of PhMs in soil vary widely, with half-lives varying from days to years. These differences are due to differences in soil properties (moisture content, OC and clay content, pH, and soil bioactivity), soil temperature, and the physicochemical properties of the substance (lipophilicity and the degree of dissociation). Adsorption and degradation are two major environmental pathways of PhMs in soil. The sorption of PhMs on soil colloids affects the mobility of PhMs and even their degradation and bioavailability in the soil [5,94,115]. The accumulation, distribution, transport, and metabolization of PhMs in soils vary depending on the surface chemistry of the soil. PhMs favorably adsorb to a specific soil type and show soil type-dependent sorption affinity, mobility, and dissipation [116]. Therefore, the soil-dependent surface of the soil is critical for predicting the bioavailability and persistence of PhMs in soils. Monteiro et al. [117] investigated the effect of chemical mixture interactions on the degradation of three pharmaceuticals (carbamazepine, naproxen, and fluoxetine). They found that, in single-compound studies, naproxen degraded in a range of soils with half-lives varying from 3.1 to 6.9 d, while carbamazepine and fluoxetine were more persistent. When degradation was evaluated using a mixture of the three studied compounds and the sulfonamide antibiotic sulfamethazine, fluoxetine and carbamazepine degraded similarly to in the single-compound studies, while naproxen degraded significantly more slowly in the mixture-spiked soils than in the single-compound studies. In a study conducted by Salvia et al. [118] that included 23 PhMs and two soils with different textures (silty clay loam and sandy loam), the majority of the compounds degraded relatively rapidly (*t*_½_ < 20 d), with the exception of roxithromycin (*t*_½_ = 57–88 d) and carbamazepine (*t*_½_ = 170–330 d).

Although there are many factors influencing the disappearance of PhMs in soil, most of the models proposed to study degradation are based on considering the concentration of PhMs as the only dependent variable. For the assessment of their persistence, knowledge of degradation kinetics is essential. The degradation of PhMs can be described by several models. Simple single-first-order (SFO) kinetics (monophasic model) remains the most common mathematical description of decay in the scientific literature, although it is not always possible to describe degradation using SFO kinetics. When a rapid initial decrease in the PhM concentration is often followed by a slower decrease, the pattern is usually referred to as a biphasic model, such as first-order multicompartment (FOMC), double first-order in parallel (DFOP), first-order sequential biphasic (FOSB), or Hoerl (H) models. Therefore, kinetic degradation experts from the European Commission-funded FOCUS group (Forum for the Coordination of Pesticide Fate Models and Their USe) proposed alternative equations for the degradation of soil organic pollutants [119], as summarized in Table 3.

Degradation and movement are the main routes responsible for the disappearance of PhMs in soil. Three processes deserve special attention: (i) photodecomposition (photochemical degradation), (ii) chemical degradation, and (iii) biological degradation (biodegradation). To study the degradation of chemicals in soils, different guidelines have been proposed by the OECD [120] and US EPA [121].

#### 4.2.1. Photochemical Degradation

**Figure 6:**
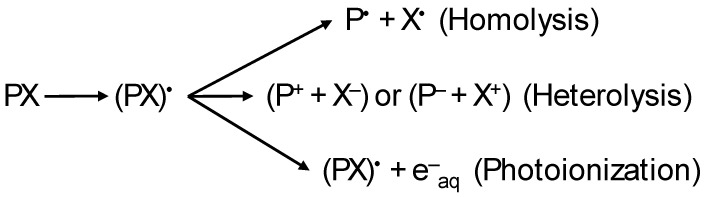
Possible direct photolysis chemical events (PX: Pharmaceutical; (PX)^•^: Excited state; P^+^, P^−^, X^+^, X^−^: Ionizated forms).

Many micropollutants, such as PhMs, are degraded by ultraviolet (UV) light. The process begins when the pollutant receives energy, which results in the excitation of electrons that can break or form less stable bonds. This occurs as long as the energy of the absorbed photons is equal to or greater than the bond energy [122]. Photolysis can be direct, where the compound absorbs UV light within the solar spectrum (<400 nm), or indirect, where the energy is absorbed by other components that subsequently transfer it to the PhM molecule or generate various reactive species. Direct irradiation promotes PhMs to their excited singlet states, which can then transition to triplet states. As shown in Figure 6, these excited states can then undergo (i) homolysis, (ii) heterolysis, or (iii) photoionization, among other processes [123].

PhMs that are photodegradable, nonvolatile, and water-soluble are particularly vulnerable to photodegradation on soil surfaces. These three properties are common to most antibiotics. It has generally been assumed that light has little effect on the degradation of PhMs in soils, in contrast to what occurs in aqueous solutions [124]. Thus, soil samples spiked with 0.5 mg kg^−1^ of two widely used veterinary fluoroquinolones (marbofloxacin and enrofloxacin) were exposed to solar light, which promoted the extensive degradation (80%) of both compounds within 60–150 h, although it was two orders of magnitude slower than in an aqueous solution [125]. In another study in which soil samples containing oxytetracycline, chlorotetracycline, sulfanilamide, sulfadimidine, sulfadiazine, sulfadimethoxine, sulfapyridine, fenbendazole, and p-aminobenzoic acid were exposed to arc light or kept in the dark for 28 d, all antibiotics underwent direct photodegradation in water, with first-order rate coefficients (*k*) fluctuating from 0.005 to 0.12 d^−1^. However, for sulfonamides and fenbendazole, the average *k* in soil was 0.01 d^−1^, which was 2.4 times lower than that in water [126]. Therefore, photochemistry could be considered an important pathway for the removal of PhMs in soil, with the presence of photocatalysts, irradiation intensity and duration, aeration and soil pH, physical state, chemical structure of the PhM, and adsorption degree on colloids being the main factors influencing the process.

#### 4.2.2. Biochemical Degradation

**Figure 7:**
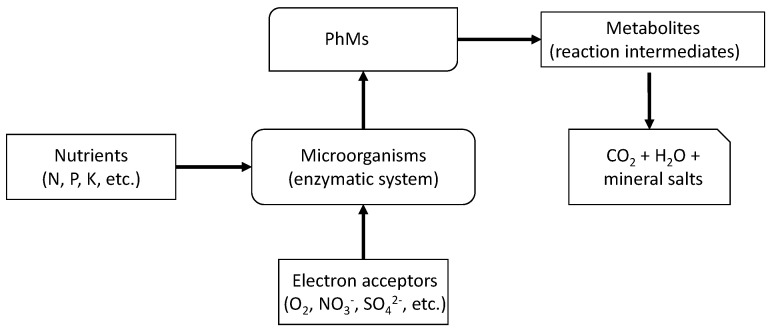
Diagram showing the biodegradation of PhMs in soil.

In principle, chemical and biological degradation can be distinguished. However, in many cases, they are closely related, and it is not easy to establish the independence of the two processes. Chemical degradation involves oxidation, hydrolysis, and other reactions that occur in soil as a function of pH, temperature, and moisture, while biodegradation can be defined as “the process by which soil microorganisms metabolically or enzymatically transform or modify the structure of PhMs present in the soil” [127] (Figure 7). To study both types of degradation (chemical and biological) separately, it is necessary to destroy soil microorganisms by appropriate irradiation or sterilization techniques. This also requires the modification of other catalytic systems that have a significant impact on degradation. For this reason, the two types of degradation are often combined and treated as biochemical degradation.

In general, the biodegradability of a PhM in an aerobic environment decreases as the molecular weight and the number of carbon atoms and aromatic nuclei in the molecule increase [122]. The process of biological degradation is slowed by (i) a lack of essential nutrients for microorganisms (e.g., nitrogen and/or phosphorus); (ii) a lack of sufficient electron acceptors (usually oxygen); (iii) a lack of appropriate environmental conditions (pH, redox potential, humidity, and temperature); (iv) a lack of microbial populations with sufficient enzymatic potential to degrade contaminants; and (v) the presence of toxic components in the contaminant mixture. While biodegradable PhMs are degraded by soil microorganisms within days or weeks, recalcitrant PhMs persist for long periods of time (years or even decades).

PhMs can be degraded by soil microorganisms (bacteria, fungi, algae, protozoa, and viruses) via aerobic processes in which oxygen acts as an electron acceptor [128] or via anaerobic processes in which nitrates, sulfates, and others act as electron acceptors [129]. Some studies have shown that the biodegradation of some anti-inflammatory drugs (clofibric acid, gemfibrozil, ibuprofen, fenoprofen, ketoprofen, naproxen, diclofenac, and indomethacin), except for naproxen, decreases poorly under anaerobic conditions [130]. The higher degradation rates under aerobic conditions suggest the possibility of an enhanced degradation of PhMs by oxygenation. Generally, lower dissipation half-lives and variability for some PhMs (i.e., atenolol, clindamycin, metoprolol, trimethoprim, and sulfamethoxazole) were found in higher-quality soils with well-developed structures, high nutrient contents, etc., and better microbial conditions (chernozems) than in lower-quality soils (Cambisols) [90]. Nonionic compounds such as carbamazepine, lamotrigine, caffeine, sildenafil, sulfapyridine, and metoprolol were recalcitrant and accumulated in soils irrigated with effluents from WWTPs containing these PhMs, whereas weakly acidic PhMs exhibited rapid degradation rates in soils, probably related to their chemical structure. However, Carr et al. [131] demonstrated that the half-lives of different PhMs (estrone, 17β-estradiol, estriol, estrogen, 17α-ethinylestradiol, triclosan, and ibuprofen) increased under water-saturated conditions compared to those in draining soil, with degradation in wet soils increasing after the third day when the soils neared field capacity after draining; however, Grossberger et al. [132] showed that the physicochemical properties of PhMs have a major influence on biodegradation kinetics, while soil properties have a minor influence.

Due to the large number of PhMs with different properties currently in use, the number of metabolites or transformation products generated by dealkylation, dehalogenation, hydrolysis, oxidation, etc., is very high [133]. These new structures can be completely degraded by mineralization to CO_2_, H_2_O, and mineral salts. Alternatively, they can be incorporated into the humic substances of soil by polymerization. This leads to the formation of other highly stable substances. These nonextractable and chemically unidentifiable fractions, which remain in the humic fractions of the soil after extraction with solvents of different polarities, are known as soil-bound residues.

### 4.3. Movement (Transport) of PhMs in Soil

Several processes can transport PhMs in the soil environment [85]: (i) diffusion (movement through the soil from a site of a higher concentration to a site of a lower concentration), (ii) volatilization (change of solids or liquids into a gas that moves through pore space), (iii) erosion and runoff (movement of soil particles exposed to transport by water on a sloping surface, wind, or organisms), (iv) bioaccumulation by organisms and plant uptake (movement of PhMs into soil organisms and plants), and (v) leaching (the movement of pollutants in water through the soil, up, down, and/or sideways).

#### 4.3.1. Diffusion

According to Fick’s law, which states that the net number of particles passing through a given area per unit of time is proportional to the concentration gradient with the opposite sign, a PhM will move through soil from an area of a higher concentration to an area of a lower concentration (Equation (3)):

(3) $$J=-D\frac{\partial \emptyset }{\partial x}$$

where *J* is the diffusive flux (mol m^−2^ s^−1^), *D* is the diffusion coefficient (m^2^ s^−1^), Ø is the pollutant concentration (mole m^−3^), and *x* is the position (m).

This is observed both in the gaseous phase of the soil and in the liquid or air phase between the solid particles. The diffusion coefficient, solubility, and vapor pressure of the PhM and, in particular, the temperature, humidity, soil porosity, and adsorption degree of the compound are the main factors influencing this process [134].

#### 4.3.2. Volatilization

A common pathway for the movement and disappearance of PhMs could be the volatilization of PhMs from the soil and their subsequent diffusion into the atmosphere. However, some studies indicate that the removal of PhMs by volatilization could be neglected due to their low Henry’s law constants [135]. Their potential volatility is closely related to their vapor pressure, but their effectiveness depends largely on the colloidal composition, structure, porosity, water content, soil temperature, and pH, as well as their type, concentration, and degree of adsorption. High temperatures favor this process unless the soil dries quickly. The soil moisture is also significant, as PhMs evaporate more quickly from wet soils than from drier soils. However, it should be noted that PhMs with physical adsorption (weak) volatilize faster than those with strong adsorption (chemical). This is because they are more susceptible to exchange with water molecules.

#### 4.3.3. Runoff

Runoff/erosion processes result in the transfer of soil from fields to adjacent land/water bodies. There are two interdependent processes involved in runoff. The first is the disruption of soil aggregates and the movement of the resulting fragments. The second is that particles from the fracture are at the mercy of transport agents (water, wind, or living organisms) once the aggregate has been destroyed [136]. Surface runoff, also known as overland flow or terrestrial runoff, occurs when an excess of rainwater, stormwater, snowmelt, or other sources of water cannot infiltrate the ground fast enough. This can occur when the soil is completely saturated with water (saturation-excess overland flow), when rainfall occurs faster than the soil can absorb it, or when the soil is unsaturated but the rate of water delivery to the surface exceeds the rate of infiltration into the soil (infiltration-excess overland flow). These processes transport plant nutrients (nitrogen, phosphorus, etc.) and micropollutants such as pesticides and PhMs. The main factors influencing runoff are (i) the volume and intensity of rainfall events, (ii) the soil type and properties, (iii) landscape factors (e.g., slope), (iv) the time since the application of the PhM, (v) the physicochemical properties of the specific compound and its adsorption degree, and (vi) soil and crop management practices and land use patterns.

#### 4.3.4. Bioaccumulation by Soil Organisms

There are many organisms in agricultural soils that degrade or absorb certain PhMs during their life cycle, which may result in higher PhM concentrations in their bodies than in the environment [137]. To assess the likelihood of the absorption and distribution of a PhM in a given organism, it is very useful to determine the partition coefficient (*K*_OW_). These values, generally expressed as a decimal logarithm (log *K*_OW_), are constant for each PhM at a given temperature. A high coefficient implies that the compound is likely to accumulate in living organisms, and the nature of binding to biological receptors also plays a role. However, a low coefficient reduces the potential for bioaccumulation, that is, the net accumulation of a PhM in an organism over time from both biotic (other organisms) and abiotic (soil, air, and water) sources [138].

#### 4.3.5. Plant Uptake

Numerous studies have shown that many crops grown in areas where PhMs are notoriously present as a consequence of WW irrigation or the addition of polluted biosolids can absorb some of these compounds from the soil in varying proportions [87,91,100,139,140,141,142,143,144,145,146,147,148,149,150]. The extent of the process depends on a number of factors, such as the type of crop, the physicochemical properties of the compound (molecular weight, vapor pressure, water solubility, and *K*_OW_), environmental characteristics (soil type, temperature, water content, and agricultural practices), and plant characteristics (root development, the shape and size of leaves, and lipid content) [151].

**Figure 8:**
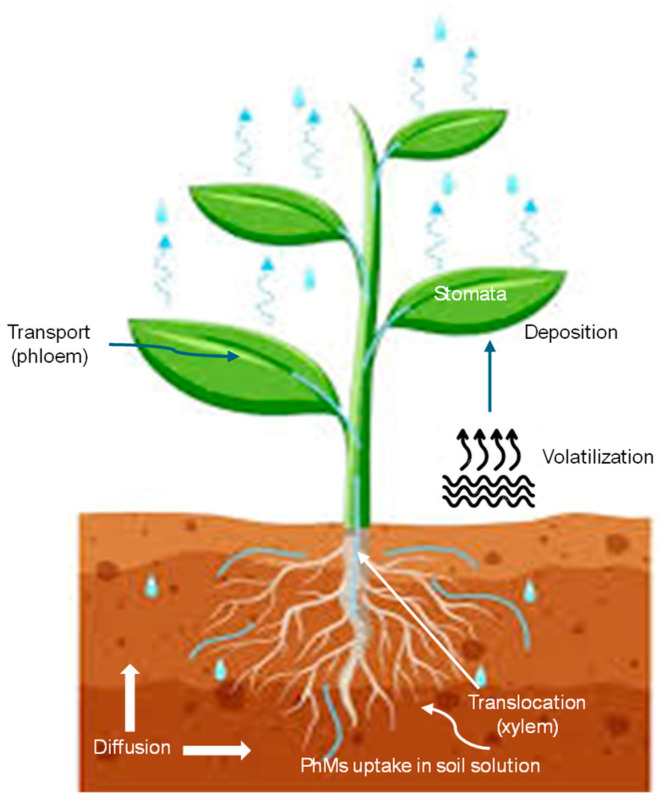
Scheme showing main plant uptake pathways.

The bioconcentration factor (BCF), which is usually calculated as the ratio of the drug concentration in the plant to that in the bulk soil, is commonly used to characterize the distribution of PhMs in soil–water–plant systems [152]. To obtain better knowledge on how soil PhMs enter plant cells, it is vital to understand the major pathways and processes involved in the uptake and translocation of these pollutants, which can be taken up by plants from the soil and its constituents through the root system. In contrast to active transport, which requires energy to transport nutrients and pollutants across the cell membrane, the movement of PhMs across the cell membrane is achieved by passive diffusion, which does not require the cell to expend energy [153]. Contaminants with high vapor pressures and high Henry’s law constants affect the pollutant uptake from the air due to their high gaseous concentrations. The major uptake pathways in plants are shown in Figure 8.

Researchers have made great strides in understanding the mechanisms that affect the plant uptake of PhMs. These include specific factors such as *K*_OW_, the charge of the chemical, and the half-life value. In assessing the potential risk to human health, the type of plant species is also important. The accumulation of many nonionic pollutants by plants is strongly related to their lipophilicity. This is indicated by the linear correlation between BCFs and *K*_OW_ [154]. However, the combination of hydrophobicity, chemical speciation, and pH determines the uptake of ionic compounds by plants. Many PhMs are ionizable and have low hydrophobicity. Hence, the relationships developed for nonionic pollutants may not be applicable to PhM uptake. Thus, no apparent relationship was observed between the log BCF and log *K*_OW_ for 20 PhMs (including acidic, basic, and neutral compounds) in hydroponically grown spinach, lettuce, pepper, and cucumber [99]. However, intense correlations were reflected when the data were restricted to neutral PhMs. Zhang et al. [155] reported that caffeine (uncharged compound) is readily taken up by aquatic plants, while diclofenac (negatively charged) is not, because plant cells have a negative cell membrane potential. This leads to the repulsion of negatively charged anions [156]. In addition to pollutant-specific pathways, contaminant uptake may vary by plant species due to the root system, leaf shape and size, transpiration rate, and lipid content. For example, root vegetables like carrots are likely to absorb more soil contaminants than tree fruits (apples), as root crops are intimately connected to the soil, whereas tree fruits are not. However, the uptake of pollutants directly from the air is estimated to be greater for tree fruits than for root crops [151].

A major consequence of soil pollution with PhMs is that the residues of these compounds may enter the food chain after plant uptake, with consequent risks to human/animal health through dietary consumption. Evidence demonstrating that plants can take up and accumulate these pollutants, not only in their roots but also in their edible parts, has raised concerns about the presence of PhMs in food crops [157], although the levels measured are generally low. The long-term effects of these compounds on human health, however, are poorly understood. Paltiel et al. [158] detected carbamazepine and its transformation products in human urine after the consumption of fresh produce irrigated with treated WW. Several reports have assessed the potential human health risk associated with the intake of foods polluted with PhMs. To estimate an individual’s annual exposure, Wu et al. [99] used data from the leafy vegetables lettuce and spinach. The annual exposures varied from 0.1 to 150 μg for lettuce and from 0.04 to 350 μg for spinach. Wu et al. [159] also calculated annual exposures to seven PhMs from the consumption of mature crops irrigated with PhM-enriched reclaimed water and found exposures of about 4 μg/person. This exposure value is much lower than that detected for a single medical dose, which typically ranges from 20 to 200 mg [160]. Other studies concluded that the concentrations of most PhMs in plant tissues pose a minimal risk to humans [161,162].

#### 4.3.6. Leaching

Transport processes can determine the fate of PhMs and the risks associated with their exposure to the environment. Under certain conditions, some PhMs can migrate through the soil profile by leaching [163,164,165,166,167,168,169,170,171,172,173]. The soil leaching of PhMs is a common process in the environment. It occurs through the action of rainwater or irrigation. It is essential that the product has sufficient water solubility for this process to occur. The transport of PhMs in soils occurs mainly via matrix flow. However, in many cases, the macropores of the soil act as a preferential pathway, resulting in the rapid movement of contaminants toward the unsaturated zone. In many European countries where a high percentage of drinking water is obtained from underground sources, the leaching process is of critical importance.

In the leaching process, the physicochemical properties of the contaminants, as well as the characteristics of the soil (texture, clay content, organic matter, and permeability), play a predominant role [174]. Among these factors, however, the *K*_OC_ content is the most important because it largely determines the adsorption of chemical contaminants and, consequently, their mobility [175]. *K*_OC_ is commonly used as an effective tool to estimate the potential mobility of pesticides and PhMs in soil. PhMs may be suspended in water, dissolved, or simply emulsified. The extent of the process depends on the nature of the compound used and, more importantly, on the colloidal soil composition and its adsorption potential, which can be partially quantified by laboratory experiments using soil columns with the compound of interest or in the field using lysimeters. The relative mobility (leaching distance) is inversely proportional to the *K_d_* in the soil. Several guidelines have been proposed by the OECD [176] and US EPA [177] to study the movement of chemicals in soils. A number of models for assessing the mobility of micropollutants through soil have been proposed in recent decades. Most of the indices published to assess leaching potential are based on adsorption and degradation as the main factors. However, there are others that include soil and environmental parameters [175].

As mentioned above, many authors have noted that the adsorption/desorption process is primarily responsible for regulating the rate and extent of PhM leaching. Therefore, when a compound is adsorbed on the organo-clay complex, it should not be affected by other processes. Therefore, a good strategy to reduce leaching is to increase the organic matter content of the soil through various agronomic practices, such as the addition of fresh or composted biosolids or the addition of plant biomass, as this will increase the adsorption of nonionic compounds [178]. It should also be noted that the addition of organic residues to agricultural soils is a usual “ecological practice” in many European countries to increase soil fertility and, consequently, productivity. The main consequence of adding OM to soils is a reduction in the mobility of contaminants, although this reduction is due not only to the additional presence of OM but also to structural changes in soil porosity as a result of the increase in OC. Quin et al. [168] investigated the mechanism that allows soil organic matter (SOM) to influence the retention and transport of two PhMs (ibuprofen and carbamazepine). The results showed that SOM could significantly influence the environmental behavior of PhMs via surface coating and pore filling, reducing the sorption of dissociated PhMs (ibuprofen) but increasing the sorption of nondissociated PhMs (carbamazepine), while pore filling with colloidal SOM improved the retention of both compounds by providing nano/micropores and limiting diffusion. The greater retention and lower mobility of these compounds in soil microaggregates than in bulk soil suggest that the SOM content and SOM-altered soil pore structure may have a linked effect on their accumulation. In addition, it should be noted that dissolved organic matter (DOM) can affect the adsorption and movement of PhMs in soil, as there may be competition between PhMs and DOM for adsorption sites, and there may also be PhM–DOM interactions that favor leaching. However, the addition of OM to soils usually means an increase in microbial activity, which increases the biodegradation of PhMs. In some cases, microorganisms may prefer to use OM as a source of carbon and energy rather than as a micropollutant [179].

After the irrigation of soil columns with contaminated WW, between 0% and 7% of ibuprofen, naproxen, and diclofenac amounts were recovered from the soil profile, while carbamazepine was more persistent (55%–107%). The levels in leachates suggested that movement through the soil was possible for all compounds, particularly in profiles with low OM and clay content [163]. Bondarenko et al. [165] irrigated mature turfgrass plots with unspiked tertiary-treated WW for more than 6 months and collected leachates at a 90 cm depth on a weekly basis. Trimethoprim and primidone were commonly detected in leachates, whereas sulfamethoxazole, meprobamate, and carbamazepine were less commonly detected (<50%). The total mean leachate concentration was 10 ng L^−1^ for trimethoprim; 7 ng L^−1^ for primidone; and 3 to 12 ng L^−1^ for carbamazepine, sulfamethoxazole, and meprobamate. Column leaching experiments showed that the addition of biosolids generally increased the retardation of PhMs, while treated WW increased the mobility of weakly acidic PhMs in soils amended with biosolids [166]. Pan and Chu [169] indicated that long periods of rainfall promoted the downward leaching of tetracycline, sulfamethazine, norfloxacin, erythromycin, and chloramphenicol in soils, with higher leachability in sandy soils than in clay or loamy soils. Compared to groundwater-irrigated vadose zone soils, WW-irrigated vadose zone soils had significantly higher PhM detection frequencies and contamination levels. This suggests the important role of irrigation water sources in PhM accumulation and transport in the vadose zone [170]. Hill et al. [171] demonstrated the presence of flunixin, 17a-hydroxyprogesterone, triclosan, and sulfadimethoxine in leachates using undisturbed soil columns. Other studies have shown a high-to-moderate mobility of tramadol and carbamazepine, as well as two transformation products, O-desmethyltramadol and 10,11-dihydro-10-hydroxycarbamazepine, in sandy soils [172].

## 5. Impact of PhMs on Soil Health

Soil health can be defined as “the continued ability of the soil to function as a vital ecosystem that supports plants, animals and humans”. To achieve climate neutrality, ensure a clean and circular economy, and prevent desertification and land degradation, healthy soils are essential. They are also essential for the reversal of biodiversity loss, the provision of healthy food, and the protection of human health. The EU Soil Strategy for 2030 provides a framework and concrete steps for protecting and restoring soils and ensuring their sustainable use. To ensure a level playing field and a high level of environmental and health protection, a new directive on soil monitoring has been proposed [180]. This proposal addresses the main threats to EU soils, including erosion, floods and landslides, soil organic matter loss, salinization, pollution, compaction, sealing, and soil biodiversity loss.

The ideas that prevailed until the 1960s about the Earth’s ability to purify itself by diluting pollutants in the soil, air, and water can no longer be accepted. It is now known that nature has mechanisms for retaining and concentrating pollutants, which, in certain cases, can cause not only significant changes in the ecological balance but also undesirable toxicological effects on the life forms that they affect. Because a variety of biological and geochemical processes occur in soils with a high degree of spatial and temporal heterogeneity, three types of indicators are used to quantify soil quality [181]: chemical, physical, and biological. Thus, different authors agree that biochemical and biological properties are the most appropriate for estimating soil quality due to their high sensitivity to changes in soil nutrients such as N, P, C, and S [182]. Enzymatic activities provide information on the microbiological state of the soil and its physicochemical properties and quickly reflect changes in soil quality. Therefore, the evaluation of microbial responses to PhMs in agricultural soils is critical for improving our fundamental understanding of the fate of micropollutants and their potential impact on the environment and human health. In addition, the positive effect of macro-organisms such as earthworms must be considered [137].

Different studies on the microbial response to PhMs in soil have focused mainly on the effects of antibiotics on microbial activity and communities, exposure to a single compound, exposure to high concentrations, exposure during a short-term incubation period (<21 days), and/or a single soil type [183,184,185,186,187,188,189]. Recent studies have shown that PhMs affect the soil microbial community by both stimulating and inhibiting microbial respiration and biomass. This suggests that microbial responses to PhM exposure in soil are diverse [190]. Frková et al. [191] focused on the immediate (1 d), short-term (13 d), and long-term (61 d) effects of six PhMs (clindamycin, sulfamethoxazole, carbamazepine, citalopram, fexofenadine, and irbesartan) on microbial communities in seven soils with different physicochemical properties. Basal respiration was used to indicate microbial activity, while phospholipid fatty acids were used to determine microbial biomass and community structure. The authors identified four microbial responses to PhMs, namely, stimulated, inhibited, stressed, and dormant, which were highly substantial in the short term. The stimulatory impact of sulfamethoxazole was the most pronounced. This change was accompanied by a shift in the structure of the microbial community in favor of fungi and G-bacteria. The inhibitory effect, with minor changes in the microbial community structure compared to an unsupplemented control, was mainly observed for citalopram, irbesartan, and a PhM mixture in Cambisol Dystric. A stress effect was observed for all PhMs in Arenosol and Cambisol Haplic, while a dormancy effect was mainly observed for most PhMs in Chernozem Siltic. The microbial responses were highly dependent on the soil type, specific PhM, and time. This highlights the importance of considering these parameters, including the resilience of soil microbial communities to micropollutants, in the long-term management of agricultural soils. Thus, when considering the resilience of soil microbial communities to PhMs in long-term agricultural soil management, determining microbial responses under different exposure conditions may help elucidate the effects of PhMs on microbial activity, community size, and structure in diverse soils.

## 6. Remediation of PhM-Polluted Soils

Remediation is a term generally used to describe the cleanup or restoration of a polluted environment. This means taking steps to prevent pollution from spreading and further degradation to a level that allows for future use, revitalization, and remediation. Therefore, the goal of remediation is to reduce the concentration of contaminants in the environment (air, water, and soil) to an acceptable level or to eliminate them completely [192]. The procedures and methods of ecological soil management, commonly called soil ecosystem management or soil conservation, are aimed at maintaining or improving soil fertility, health, and general quality. There are three areas of equity that should be considered in ecological soil management: (i) environmental equity, (ii) scientific equity, and (iii) socioeconomic equity.

Restoring degraded soils is one of the best approaches that can be used to improve soil quality and support sustainable agriculture while contributing to long-term environmental protection [193]. Thus, ecological management is aimed at maintaining sustainability. This refers to maintaining, conserving, and improving soil quality and production while reducing environmental degradation to ensure long-term health and productivity. The process of minimizing, cleaning, or restoring soils that have been damaged by stressors such as pollutants, invasive species, or other types of soil degradation mechanisms is referred to as soil restoration [194].

Sustainable soil remediation has been defined by the International Organization for Standardization (ISO) working group as an approach that minimizes the overall negative environmental, social, and economic impacts of remediation activities while eliminating and/or controlling unacceptable risks in a timely and safe manner [195]. The ISO paper concludes the following: “The need for remediation is identified through risk assessment, and the process of selecting the remediation strategy involves determining the feasible plan that would provide the best overall environmental, social, and economic benefits from the remediation effort” [196].

Soil remediation is a critical process whose goal is the return of contaminated soil to a clean and healthy state. Various techniques and strategies are used to mitigate the presence of PhMs and other harmful pollutants to ensure the protection of human health and the environment. Soil remediation is critical when soil contaminants pose a threat to the environment. They can leach into groundwater and surface water, affecting the entire ecosystem. Contaminated soil is a major challenge to agricultural productivity. By remediating contaminated farmland, we can ensure the production of safe and healthy food, protect crop yields, and support sustainable agricultural practices. In addition, by remediating contaminated soil, we can prevent the further spread of contaminants and protect sensitive habitats, flora, and fauna.

Traditional methods of soil pollution management have relied on the isolation of affected areas and the use of containment barriers to prevent potential leaks [197]. While these methods are effective in preventing the spread of toxic substances in soil, they are a temporary solution because they do not address the source of the contamination. Since the beginning of this century, new technologies have been developed for the decontamination and reuse of contaminated soils, called remediation techniques, which include a series of operations that modify the structure of the contaminants through chemical, physical, or biological actions to reduce the toxicity, mobility, or volume of the polluted material. It should be noted that the selection of the appropriate remediation technique, as well as the design and strategy of the process, determines the success of a decontamination process. However, to make this selection, certain factors must be considered. Each situation is different and must be studied in detail. Below are the main criteria that have an impact on the choice of remediation technique:

- Environmental characteristics. The topography, demography, hydrology, and ecology of the contaminated area.
- The type, concentration, and toxicity of the contaminant. The type of pollutant (organic or inorganic) and its physicochemical characteristics provide us with necessary information on the behavior of the pollutant in the soil and its greater or lesser persistence and hazardousness.
- Physicochemical properties and type of soil to be treated. The texture, structure, porosity, permeability, heterogeneity, pH, temperature, humidity, and OM content are the parameters that determine the choice of one technique or another.
- Cost of the technique. The inherent uniqueness of each pollution event makes it difficult to rigorously compare the costs of different remediation techniques. The data on which we can rely are based on a treatment applied under specific conditions, and it can be very difficult to extrapolate to other conditions with different contaminants at different concentrations and with different types of soil. In addition, these costs can be expressed as different parameters/units (the volume of soil treated, reduction in pollutant concentration, reduction in pollutant mobility, mass of pollutant removed, or area treated), which increases this difficulty. In general, thermal techniques are the most expensive, and biological techniques are the most economical.

**Figure 9:**
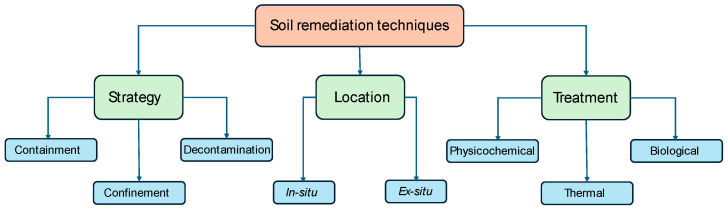
Soil remediation techniques depending on the strategy used, the location of the process, and the type of treatment.

Remediation techniques can be classified based on three criteria: (i) the remediation strategy, (ii) the place where the remediation process is carried out, and (iii) the nature of the treatment (Figure 9).

Depending on the strategy selected, we can distinguish three basic methodologies that can be used, individually or together, to remediate contaminated soils:

- The immobilization or isolation of contaminants.
- The separation or extraction of contaminants.
- The destruction or transformation of pollutants

Depending on the place where the treatment is performed, two types of techniques can be differentiated:

- In situ: When the treatment is carried out directly on the contaminated area, there is no need to excavate the site. These are more economical techniques because the soil does not have to be excavated or transported. These methods have the disadvantages of requiring longer treatment times, having a heterogeneous distribution of contaminants in the soil, and having difficulty verifying the effectiveness of the processes.
- Ex situ: When a process (dredging, excavation, etc.) is necessary to remove (move/transport) the contaminated area before its treatment, it can occur on site or in a different place (off-site). Among the advantages of these techniques, we can mention shorter treatment times and greater uniformity in the soils to be treated, as they can be homogenized periodically. In contrast, some equipment is needed to excavate the soil, which makes the process more expensive. In addition, there are risks associated with handling the material and possible exposure to the contaminant.

Finally, depending on the type of treatment, three methods can be used:

- Bioremediation is a natural and environmentally friendly approach that uses microorganisms or plants to break down or neutralize contaminants in soil. Microorganisms, such as bacteria and fungi, are able to metabolize contaminants and convert them into less harmful substances. In a process known as phytoremediation, plants can absorb and accumulate contaminants. For the removal of PhMs in soils, several plant species have been highlighted, such as *Salix exigua* (7α-ethynylestradiol); *Helianthus annus* L. (tetracycline and oxytetracycline); *Softstem bulrush* (caffeine, naproxen, diclofenac, carbamazepine, and clofibric acid); *Brassica nigra* (aspirin and tetracycline); *Typha latifolia*, *Phragmites*, *Iris germanica*, *Juncus effuses*, and *Phragmites australis* (ibuprofen); *Lemna gibba* (lomefloxacin, sulfamethoxazole, and chlortetracycline); or *Glycine max* (carbamazepine, diphenhydramine, fluoxetine, triclosan, and triclocarban) [198]. Bioremediation is effective for organic contaminants, including petroleum hydrocarbons, solvents, pesticides, and pharmaceuticals.
- Chemical remediation involves the use of chemicals or chemical processes to treat soil contamination. The chemical composition of pollutants is altered to make them less toxic or immobile using techniques such as oxidation and reduction reactions. Soil washing, soil vapor extraction, and chemical oxidation are common chemical remediation methods. A wide range of persistent organic pollutants are amenable to these methods.
- Physical remediation techniques focus on the physical removal or isolation of contaminated soil from the surrounding environment. A common approach to physical remediation, particularly for localized contamination, is to excavate and remove contaminated soil. Soil capping is another method in which contaminated soil is covered with a barrier to prevent further contamination. Physical remediation is often used for soils that have been contaminated with hazardous materials, such as asbestos or radioactive materials.

Therefore, the problem of soil decontamination can be treated from two fundamental perspectives: (i) contaminant isolation techniques and (ii) decontamination techniques. The dilemma that arises in the face of contaminated soils is whether to recover or destroy them. Recently, special attention has been given to recovery techniques that make it possible to reuse them as opposed to traditional isolation techniques [192,199,200]. The main techniques used are summarized in Table 4.

## 7. Conclusions

Among the major types of EPs, PhMs are one of the groups of greatest interest due to their widespread use worldwide and their impact on the environment. However, a discussion of the achievements in the field of drug therapy should not overlook the many unresolved problems of drug residues in the environment.

PhMs are often found in significant concentrations in soils due to the continuous release of effluent and sludge from WWTPs, which is substantially faster than their removal rates. In addition, the land application of animal manure can lead to the contamination of soil, surface water, and groundwater with PhMs through surface runoff and leaching. Reclaimed water is increasingly being used to supplement water resources in arid and semiarid zones. However, this water has a complex matrix that includes PhM residues, among other EPs, which are introduced into the soil when it is used for irrigation. In addition, the use of SS as an organic amendment in soils can contribute pollutants to the soil. European policy is directly aimed at increasing the use of treated WW for crop irrigation and the agricultural reuse of SS in soil to improve its fertility. However, the impacts of long-term application on soil properties are still unknown. Although PhMs are generally present at low environmental concentrations, it remains unclear whether their presence in terrestrial and aquatic environments can cause humans or wildlife to be adversely affected.

The behavior and fate of PhMs in soils are governed by several processes, with adsorption determining how PhMs degrade and move. In this regard, the colloidal components of the soil, both inorganic (clays) and organic (humus), play a fundamental role. The sorption of PhMs by soil particles generally reduces their uptake by plants, especially for compounds with strong hydrophobicity or a positive charge. However, numerous studies have shown that many crops grown in areas where PhMs are notoriously present can absorb some of these compounds from the soil in varying proportions. The extent of the process depends on a number of factors, such as the type of crop, the physicochemical properties of the compound, environmental properties, and plant characteristics. A major consequence of soil pollution with PhMs is that the residues of these compounds, when taken up by plants, can enter the food chain and pose potential human/animal health risks when ingested.

In addition, recent reports suggest a diversity of microbial responses to PhM exposure in soil, indicating that PhMs affect soil health by altering soil microbial communities through both the stimulation and inhibition of microbial respiration and biomass. In view of the above, further measures are needed to prevent soil contamination caused by PhMs. Measures are also needed to clean up and remediate soil contamination in order to improve its subsequent reuse while protecting the health of animals and humans.

## Figures and Tables

**Table 1:** Chemical structures and physicochemical properties of some of the main PhM compounds.

Therapeutic Class	Compound	Structure	Log *K_OW_*	*pK_a_*	Water Solubility (mg L^−1^)
Analgesics and anti-inflammatories	Acetylsalicylic acid	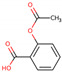	1.2	3.5	4600
	Dicoflenac	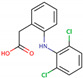	4.5	4.1	2.4
	Ibuprofen	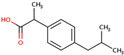	4.0	4.9	21
	Naproxen	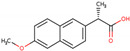	3.2	4.1	16
Antibiotics	Azithromycin^MC^	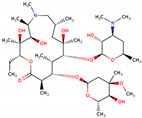	4.0	8.7	7.1
	Clarithromycin^MC^	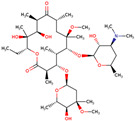	3.2	9.0	0.3
	Erythromycin^MC^	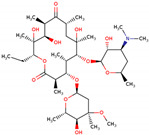	3.1	8.9	1.4
	Amoxicillin^PN^	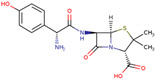	0.9	4.4	3430
	Sulfamethoxazole^SF^	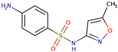	0.9	3.8	610
	Ciprofloxacin^QN^	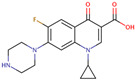	0.3	6.1	3 × 10^4^
	Oxytetracycline^TC^	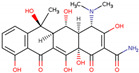	−0.9	3.3	313
Antiepileptics	Carbamazepine	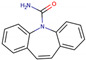	2.5	2.3	17.7
Antimicrobials	Triclosan	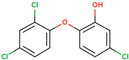	4.8	8.7	1.0
Antineoplastic agents	Cyclophosphamide	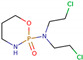	0.63	7.6	4.0 × 10^4^
β-blockers	Atenolol	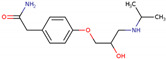	0.16	9.4	1.3 × 10^4^
	Propanolol	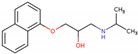	3.5	9.4	23
Hormones	17α-ethinylestradiol	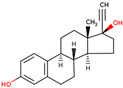	3.7	12.2	4.3 × 10^−5^
	Progesterone	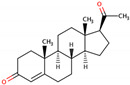	3.9	9.8	8.8
	Testosterone	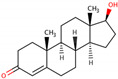	3.3	9.7	23
Illicit drugs	Benzoylecgonine (cocaine metabolite)	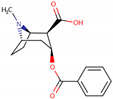	1.6	4.4	3820
	Tetrahydrocannabinol (THC-COOH) (cannabinoid)	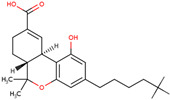	4.2	4.2	Very low
Lipid regulators	Lovastatin	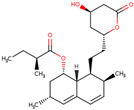	4.3	-	2.1
	Clofibrate	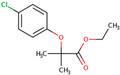	3.6	-	Very low
SSRIs	Fluoxetine	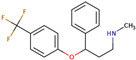	4.0	-	3.5
	Paroxetine	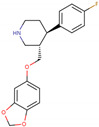	3.9	-	8.5

MC: macrolide; PN: penicillin; QN: quinolone; SF: sulfonamide; TC: tetracycline; *K_OW_*: octanol–water partition coefficient.

**Table 2:** Environmental behavior of PhMs as a function of their physicochemical properties [88].

Parameter	Values	Comments
Water solubility (mg L^−1^)	<50	Low
	50–500	Moderate
	>500	High
Hydrolysis (TD_50_, days)	<30	Nonpersistent
	30–100	Moderately persistent
	100–365	Persistent
	>365	Very persistent
Vapor pressure (*P*_V_, mPa)	<5	Low volatility
	5–10	Moderately volatile
	<10	Highly volatile
Henry’s law constant (*H*, Pa m^3^ mol^−1^)	>10^2^	Volatile
	10^−1^–10^2^	Moderately volatile
	<10^−1^	Nonvolatile
Octanol–water partition coefficient (log *K*_OW_)	<2.7	Low bioaccumulation
	2.7–3.0	Moderate bioaccumulation
	>3	High bioaccumulation
Organic carbon partition coefficient (log *K*_OC_, mL g^−1^)	<1.2	Very mobile
	1.2–1.9	Mobile
	1.9–2.7	Moderately mobile
	2.7–3.6	Slightly mobile
	>3.6	Nonmobile
Dissociation constant (*pK_a_*)	pH < *pK_a_*	Neutral state
	pH > *pK_a_*	Negative charge
GUS (Groundwater Ubiquity Score) Index	>2.8	High leachability
	2.8–1.8	Transition state
	<1.8	Low leachability

**Table 3:** Kinetic models for PhM degradation.

Model	Equation	Parameters	Endpoints
SFO	$C_{t}=C_{0}\;e^{-kt}$	2 (*C*_0_, *k*)	${DT}_{X}=\frac{ln\left[\frac{100}{\left(100-X\right)}\right]}{k}$
FOMC	$C_{t}=C_{0}{\left.\left(1+\frac{t}{\beta }\right.\right)}^{\alpha }$	3 (*C*_0_, *α*, *β*)	${DT}_{X}=\beta \;{\left(\frac{100}{100-X}\right)}^{\left(\frac{1}{\alpha }\right)}-1$
DFOP	$C_{t}=C_{1}e^{-k_{1}t}+C_{2}e^{-k_{2}t}$	4 (*C*_1_, *C*_2_, *k*_1_, *k*_2_)	*DT_x_* values can only be calculated via an iterative procedure
FOSB	$C_{t}=C_{0}\;e^{-k_{1}t}$ when *t* ≤ *t_b_*	4 (*C*_0_, *k*_1_, *k*_2_, *t_b_*)	${DT}_{X}=\frac{\ln\frac{100}{100-X}}{k_{1\;}}$ when *DT_x_* ≤ *t_b_*
	$C_{t}=C_{0}\;e^{-k_{1}t_{b}}\;e^{-k_{2}(t-t_{b})}$ when *t* > *t_b_*		${{DT}_{X}=t}_{b}+\frac{\left(\frac{\ln100}{100-X}\right)-k_{1}t_{b}}{k_{2}}$ when *DT_x_* > *t_b_*
H	$C_{t}=C_{0}e^{-kt}t^{a}$	3 (*C*_0_, *k*, *a*)	*DT_x_* values can only be calculated via an iterative procedure

*C_t_* = concentration at time *t*; *C*_0_ = concentration at *t* = 0; *k* = rate constant; *t* = time since application; *C_t_* = total amount of pollutant at time *t*; *C*_1_ = amount of pollutant in compartment 1 at time = 0; *C*_2_ = amount of pollutant in compartment 2 at time = 0; *k*_1_ = rate constant in compartment 1; *k*_2_ = rate constant in compartment 2; *α* = shape parameter determined by coefficient of variation of *k; β* = location parameter; *t_b_* = breakpoint (time at which the rate constant changes); *a* = parameter that indicates the deviation from the single-phase model (if *a* ≠ 0, the kinetic behavior will be biphasic); *DT* = disappearance time.

**Table 4:** Summary of the main techniques used in soil decontamination.

Treatment		Technique	Application
Removal	Physicochemical	Aeration	In situ
		Washing	Ex situ
		Dragging	In situ
		Adsorption	In situ
		(Photo)chemical oxidation	In situ
		Electrokinetic treatment	In situ
	Biological	Bio-augmentation and bio-stimulation	In situ
		Biopiles	Ex situ
		Composting	Ex situ
		Phytoremediation	In situ
		Landfarming	Ex situ
		Bioventing	In situ
		Natural attenuation	In situ
		Enzymatic degradation	In situ
		Biosorption	In situ
	Thermal	Incineration	Ex situ
		Thermal desorption	Ex situ
		Plasma	In situ
	Solar	Solarization	In situ
		Biosolarization	In situ
Containment and confinement		Barriers	In situ
		Deep sealing	In situ
		Solidifying injection	In situ
		Vitrification	In situ

## Data Availability

The data presented in this study are available in this article.
